# Discriminating between rival biochemical network models: three approaches to optimal experiment design

**DOI:** 10.1186/1752-0509-4-38

**Published:** 2010-04-01

**Authors:** Bence Mélykúti, Elias August, Antonis Papachristodoulou, Hana El-Samad

**Affiliations:** 1 Life Sciences Interface Doctoral Training Centre, University of Oxford, Wolfson Building, Parks Road, Oxford, OX1 3QD, UK; 2 Department of Statistics, University of Oxford, 1 South Parks Road, Oxford, OX1 3TG, UK; 3 Control Group, Department of Engineering Science, University of Oxford, Parks Road, Oxford, OX1 3PJ, UK; 4 Oxford Centre for Integrative Systems Biology, Department of Biochemistry, University of Oxford, South Parks Road, Oxford, OX1 3QU, UK; 5 Department of Biochemistry and Biophysics and California Institute for Quantitative Biomedical Research, University of California, San Francisco, 1700 4th Street, San Francisco, CA 94158, USA

## Abstract

**Background:**

The success of molecular systems biology hinges on the ability to use computational models to design predictive experiments, and ultimately unravel underlying biological mechanisms. A problem commonly encountered in the computational modelling of biological networks is that alternative, structurally different models of similar complexity fit a set of experimental data equally well. In this case, more than one molecular mechanism can explain available data. In order to rule out the incorrect mechanisms, one needs to invalidate incorrect models. At this point, new experiments maximizing the difference between the measured values of alternative models should be proposed and conducted. Such experiments should be optimally designed to produce data that are most likely to invalidate incorrect model structures.

**Results:**

In this paper we develop methodologies for the optimal design of experiments with the aim of discriminating between different mathematical models of the same biological system. The first approach determines the 'best' initial condition that maximizes the *L*_2 _(energy) distance between the outputs of the rival models. In the second approach, we maximize the *L*_2_-distance of the outputs by designing the optimal external stimulus (input) profile of unit *L*_2_-norm. Our third method uses optimized structural changes (corresponding, for example, to parameter value changes reflecting gene knock-outs) to achieve the same goal. The numerical implementation of each method is considered in an example, signal processing in starving *Dictyostelium *amœbæ.

**Conclusions:**

Model-based design of experiments improves both the reliability and the efficiency of biochemical network model discrimination. This opens the way to model invalidation, which can be used to perfect our understanding of biochemical networks. Our general problem formulation together with the three proposed experiment design methods give the practitioner new tools for a systems biology approach to experiment design.

## Background

Mathematical modelling has become an indispensable tool for modern systems biology [1,2]. Simple qualitative descriptions are proving increasingly insufficient for understanding the intricate dynamical complexity of biological phenomena. As a result, quantitative mathematical models are now routinely used in order to describe and analyze the complex dynamics generated by protein interactions [3], metabolic pathways [4,5], regulation of gene expression [6], and other biochemical processes.

A successful modelling effort is necessarily an iteration between model analysis and experiments. Testing the appropriateness of a mathematical description of any physical process should be done against experimental data, but at the same time, models should inform the design of new experiments. Traditionally, experiments have been designed using heuristic approaches: experience, intuition, or simple causal analyses. Evidently, such heuristically designed experiments are not always maximally informative, a great impediment given the cost and effort involved in the development of new measurement techniques and the implementation of standard experiments. As a result, it is becoming increasingly necessary to systematically design more rigorous and predictive experiments, in order for the iterative process involving computational modelling to result in reliable models.

To date, the majority of studies addressing experiment design for biological networks has adopted a system identification approach. In this context, experiments are designed such that the resulting data are most informative about model structure or parameter values - see, for example, [7] and [8-10], respectively. Several groups considered statistically-orientated frameworks for optimal structure identification [11] or for parameter identification [11,12]. These approaches aim to find the weighted least squares of differences between data and model prediction and make use of the Fisher Information Matrix and the associated notions of A-, D-, and E-optimality. In this framework, Yue *et al*. [13] examine optimally designed parameter estimation methods that are robust to model uncertainties (robust experiment design).

In numerous practical situations, accumulated biological knowledge about a system of interest can constrain the set of plausible model structures. In this case, one can enumerate a finite set of network topologies, closely corresponding to concrete biological hypotheses. Experiment design in this context would aim for the efficient discrimination between these well-defined alternative models; in more concrete terms, several mathematical models, corresponding to the different network topologies, can describe the behaviour of this system, within error bounds reflecting uncertainty in the data due to the experimental environment and inaccuracies of measurements [14]. Discriminatory experiment design and model invalidation can then be used to differentiate between them.

This is because mathematically, one can never validate a model [15]. At best, a model will be capable of explaining all the available data and can be tested against some of its predictions. Therefore narrowing down on the correct model can only be done from the other direction through invalidation, in order to systematically 'cross out' incorrect models. This results in an iterative cycle of system modelling, experiment design and subsequent model performance analysis that systematically proposes and then invalidates models that cannot represent the behaviour of the system. In order to optimally discriminate between candidate models, the experiments need to be carefully designed and implemented to produce new data that can be used to invalidate a seemingly good but incorrect model.

Various aspects of model discriminatory experiment design have been addressed in the literature. Bardsley *et al*. [16] investigated the problem of how measurements should be spaced in time to perform an optimally discriminating experiment between two models, and how many of them are required. More specifically, they compared different patterns of measurement spacings (geometric versus uniform spacing). Chen and Asprey [17] developed statistical approaches to parameter estimation, the assessment of model fit, and model discrimination, assuming that the response variables are uncertain. In this framework, model discrimination is based on a Bayesian approach, which assigns prior 'goodness' probabilities to each model, updates these after each experiment and chooses the model with the likelihood that has become sufficiently large compared to others. An alternative frequentist method uses repeated hypothesis tests to reject models one by one. Donckels *et al*. [18] separated the uncertainty of the model predictions and the uncertainty of the measurements and used these to design the next experiment such that it is most informative. As opposed to the traditional approach, here the expected information content of the newly designed experiment is also taken into account (anticipatory design) in order to assess the uncertainties more accurately. Kreutz and Timmer [19] gave a review of approaches to parameter estimation and model discrimination (discussing the Akaike Information Criterion, the likelihood ratio test, and alternative forms of the sum of squared differences between two models' outputs). They also discussed relevant classical statistical aspects of experiment design, such as randomization, replication, and confounding. Tidor and co-workers [20] developed dynamic model-based controllers that drive the output along a prescribed target trajectory (usually a constant output). If such a control input signal achieves the required output trajectory in an experiment, then the model is more accurate than another model which gives a different output trajectory for this particular input. In [21], Kremling *et al*. presented three methods for optimal pairwise discriminating experiment design, and compared them on a test example. Their first method compares combinations of certain initial input levels and subsequent changes in input in order to determine which combination will lead to the largest difference in the outputs. Their second method replaces models with their linearized counterparts in order to find a sinusoidal input with a frequency that maximizes the difference between phase shifts of the two models. Their third method follows the work in [17], and aims to find an input profile that brings the output responses of the two models as far apart as possible. The distance is measured by a weighted objective function. The weighting is set up such that if the measurement error of an output variable is large, then the difference of these outputs contributes less to the weighted objective function. The authors concluded that the most appropriate method strongly depends on the possible ways to stimulate the system and the quality of the measurements.

In our approach to data-supported computational modelling of biological networks, we take the view that one should follow an iterative procedure that includes model identification (model fitting), model discrimination (in which a new experiment is designed) and model invalidation (using the new experimental data). All three tasks present serious challenges, and remain important areas of research and investigation. In this paper we address the problem of model discrimination. Specifically, we present a framework for defining and designing optimally discriminating experiments, that is, experiments that are the best (in some mathematically defined but practically meaningful way) at discriminating between rival models. There are cases when it is difficult or even impossible to distinguish between rival models due to the incomplete observability of their internal states. Tests exist to identify such cases [22]. Even when model discrimination is possible, it can be expected to be difficult as the starting assumption is that the rival models both describe all available data well.

Our key principle is to maximize the difference between the outputs of two different models, in particular, the *L*_2_-norm of the output difference. Although similar in principle, our investigation follows a direction distinctly different and more practical from the work in [17]: we use deterministic models that do not take account of measurement noise directly. Instead, we try to make the outputs of the two models as distant as possible to ensure that even a noisy measurement has a good chance of discriminating between them.

We propose three approaches to achieve this goal. In the first approach, the *Initial condition design for model discrimination*, we find the initial state of the system which results in the most discriminating output between the two examined models [23]. The second method, *Input design for model discrimination*, assumes the possibility for external stimulation during the experiment and searches for the best such stimulus from a set of allowable stimuli. This approach is reminiscent of but different from the second method in [21] - there, the difference between phase shifts is maximized, whereas in our method the difference between amplitudes is maximized. The third method, *Design of structural changes for model discrimination*, combines optimal initial condition choice with optimal systemic modifications. The latter reflects the assumption that in the experiment it is possible, for example, to up-or down-regulate the expression of certain genes, either through genetic manipulations or other techniques such as RNAi technology. The gene product may be an enzyme whose concentration is not explicitly modelled but is reflected in a chemical rate constant, or some protein which exists in (possibly various) phosphorylated and dephosphorylated forms such that the sum of their concentrations is constant. In our mathematical model this means a free choice in some parameter values within given intervals. In all three approaches, we cast the problem in an optimization framework and use the *sum of squares *(SOS) technique [24] for the experiment design, allowing us to treat the nonlinear system descriptions directly. The theoretical results are demonstrated by the application of each method to a discrimination problem for two models of signal processing for chemotaxis in *Dictyostelium *amœbæ.

## Results and Discussion

### Problem formulation

In this work, we consider different models describing the same biological system by a set of ordinary differential equations using, for example, mass action, generalized mass action, or Michaelis-Menten kinetics. In general, the *i*th model takes the form(1)

where *u *is a *q*-dimensional vector denoting the input, *x*_*i *_is an *n*_*i*_-dimensional vector denoting the state, *y*_*i *_is an ℓ-dimensional vector denoting the output (which is of the same dimension for each model) and *g*_*i *_is matrix-valued, with size *n*_*i *_× *q*. The structure of the functions *f*_*i*_, *g*_*i *_and *h*_*i *_will depend on the modelling framework in use to describe the biological system, but we assume that all of them are smooth. Here, the output function represents measurements an experimenter obtains from the system, and the input function represents the stimuli or perturbations the experimenter could introduce to the system during the experiment. For mathematical simplicity we assume that the input does not affect the output directly.

In this paper our aim is to discriminate between two models of the form (1), which have *n*_1 _and *n*_2 _state variables, respectively. As these two models represent the same underlying biological system, we require that they both generate the same steady states and fit already available experimental data: our aim is to design the next experiment that will allow their discrimination. A natural way to formulate the discrimination problem is to concatenate the two models and generate the difference between their outputs:(2)

We call an experiment *optimal *if the difference between the outputs of the two models (*y*_1 _- *y*_2_) is maximal over a set of experimental perturbations of bounded 'size'. In technical terms, we aim to pick the best point in a set of allowable perturbations of the initial state conditions (*Initial condition design for model discrimination*), the set of inputs *u *(*Input design for model discrimination*), or the set of some admissible parameter changes and the set of common initial conditions (*Design of structural changes for model discrimination*) in order to maximize the so-called *L*_2_-distance between the outputs of the two rival models:

To facilitate interpretation, we implement a change of coordinates that places the investigated steady state at zero in both models. We assume that the outputs are identical in this common steady state, now the origin: *h*_1_(0) = *h*_2_(0). Throughout this paper it is also assumed that the examined steady state is asymptotically stable in both models in (2).

Since experiments must be implemented in finite time, we require that the designed input *u *be zero after some future time. For convenience, we sometimes relax this requirement and only assume that *u *is 'very small' after a certain time. Clearly, since there is only one experimental setup in reality, the input *u *must be identical for the two models.

In the case of linear systems, the description of the concatenated system (2) becomes(3)

with

where the above matrices are of appropriate dimensions. We assume that all eigenvalues of both *A*_1 _and *A*_2 _have negative real parts (we call these matrices *Hurwitz*), hence they define asymptotically stable systems. This makes *A *Hurwitz too.

### Initial condition design for model discrimination

Many biological experiments drive a cellular system into an informative out-of-equilibrium state (e.g. heat shock, osmotic shock, chemical stimulus), and then glean information from the patterns of return to equilibrium in the absence of an input. In an optimization formulation, this amounts to searching for normalized initial conditions *x*_1_(0) = *x*_2_(0) for the two models one wishes to discriminate between, that maximize the output difference ||*y*||_2 _- where *y *is defined in (2) - for the unforced system (*u *= 0). Here, we assume that the two alternative model representations of the system are written in terms of the same chemical species, thus *n*_1 _= *n*_2 _= *n*.

#### Linear case

If *x*_1_(0) is not required to be equal to *x*_2_(0), then the solution can be borrowed from standard results in linear systems theory. In particular, the optimal direction for the initial value of (3) can be found by the following procedure.

1. Find a positive semidefinite matrix, *P *≥ 0, that solves the so-called observability Lyapunov equation

The solution *P *is called the observability gramian [25].

2. Find the normalized eigenvector  corresponding to the largest eigenvalue  of *P*, that is, for  find  such that

Indeed, the direction *x*(0) =  gives the maximum output energy, since the output energy is given by(4)

However, this computation is not satisfactory since an experimentally meaningful initial condition should satisfy *x*_1_(0) = *x*_2_(0) = . To enforce this condition, we can partition *P *into blocks of size *n *× *n*,

With this decomposition, the optimal initial state is the unit norm eigenvector  corresponding to the largest eigenvalue of the matrix

To see this, substitute *x*(0) =  in (4) to get:

Hence  is maximized exactly when  is the eigenvector corresponding to the largest eigenvalue of 

#### Nonlinear case

The ideas behind model discrimination in the linear case can be generalized for application to nonlinear systems. However, we cannot explicitly compute the exact difference in the outputs of the two rival models ||*y*||_2_. Our approach avoids simulations and concentrates on finding an upper bound on ||*y*||_2 _using so-called *storage functions *[26,27] and *sum of squares *algorithmic relaxations of the resulting optimization problem.

To determine an upper bound on ||*y*||_2 _for system (2), suppose there exists a continuously differentiable function *S*: ℝ^2*n *^→ ℝ satisfying(5)

where *D *is a neighbourhood of the steady state defined by:(7)

Here we assume that *D *does not include states which are not physically meaningful, and the value of *α *will ensure this. This implies that the system is dissipative with supply rate -*h*(*x*)^*T*^*h*(*x*). Suppose that the system is released from an initial state *x*(0) inside the largest level set of *S *that fits into *D*, so that ||*x*_1_(0)||_2 _= ||*x*_2_(0)||_2 _= *β *≤ *α*. In this case, integrating condition (6) and using , we get

If we let *T *→ ∞, then(8)

by the nonnegativity of *S*. This implies that  ≤ *S*(*x*(0)), since condition (6) is valid within the whole region *D *and level sets of *S *are invariant. Hence we have found a way to bound , which involves constructing the function *S*.

It is worth noting that the result from the linear and nonlinear cases have a similar purpose. Whereas in the linear case the result is rooted in a Lyapunov equality and provides optimal solution, in the general nonlinear case one has to be content with an estimate given by inequality (8).

A condition missing from the above construction is that the two system models should be released from the same initial state. Hence, following our discussion from the linear case, one has to construct an appropriately modified *S*, . In the linear case, the desired initial conditions correspond to those that maximize the quadratic form . That is, the optimal direction was that of the eigenvector corresponding to the largest eigenvalue of matrix *R*. This is also exactly the direction corresponding to the smallest semi-axis of the ellipsoid  = *r *for some *r *> 0. In other words, we were looking for the infimum of *γ *> 0 for which the set

Similarly to the linear case, we will use a geometric argument to achieve initial condition design in the nonlinear case. Here, () plays the role of the quadratic form , and one can now decrease *γ *> 0 from infinity until the shrinking level set  touches *D' *= {*x *∈ ℝ^2*n*^| ||*x*_1_||_2 _≤ *β*, ||*x*_2_||_2 _≤ *β*}. Therefore, we need to solve the following optimization problem:(9)

The sequence of results presented so far asserts that the presence of a function *S *with the properties delineated above provides an upper bound on the energy of the difference of the outputs of two rival models.

This information can be exploited to generate experimental initial conditions that drive the system towards this bound. These methods, however, do not prescribe how one would go about finding such a function. Constructing a nonnegative function is in general a difficult problem. However, recent advances in the theory of *sum of squares *provide a computationally tractable way to relax this problem [24]. In a nutshell, instead of searching for a general nonnegative function, we can constrain our search to functions that can be parameterized as sums of squares of polynomials. Within this class, the problem can be solved through semidefinite programming, with worst-case polynomial-time algorithms (see Methods, sections A and B).

Therefore, our strategy to find a near optimal initial state for the nonlinear model discrimination is a two-step process. First, we construct an SOS function *S *that satisfies (5-6). In the second step, we search for the direction in which  is maximal, that is, we solve the optimization problem (9) (see Methods, B).

### Input design for model discrimination

A powerful approach to discriminate between two plausible models of a biological process is to design an experimental input that maximally differentiates between the dynamical behaviours of their outputs. If this input generates qualitatively different patterns in the model outputs, then one can subject the actual physical system to this designed input and then eliminate the model which differs from this pattern. The most general form of this optimally discriminating input problem is the following:

Here we assume that we are designing one input and measuring one output. We also assume that the input is of unit-energy. This can be done without a loss of generality as one can scale the equations accordingly, depending on the amount of input (ligand) available and the properties of the system under study. Our goal is to maximize the difference between the two model outputs over a transient period after application of the new input. Recall that the two models describe currently available data equally well, so that for the same (basal) input they have the same pre-stimulus steady states and the same outputs.

Solving the general optimization problem in order to generate the maximally informative input is computationally challenging. In fact, even the first order condition of optimality is a 2(*n*_1 _+ *n*_2_)-variable differential equation with boundary conditions at both ends of the time interval [28]. For that reason, our strategy will be based on approximating a maximally-discriminating input using a linearization of the system in (2), and then assessing its suitability for the nonlinear system by comparing the value it achieves to the supremum of the output difference *L*_2_-norm over the set of possible inputs. This supremum will again be computed using an SOS decomposition approach. The benefits of this strategy reside in the fact that we can use established, simple methods to find an input that gives the maximal *L*_2_-norm output for the linearized system. This (possibly suboptimal) input can then be applied to the nonlinear system, and an assessment (see below) made about how the realized output *L*_2_-norm compares to the optimal, maximally discriminating *L*_2_-gain.

#### Designing an input profile using linearization

Designing an input profile for optimal discrimination using linearization is more appropriately addressed in the frequency domain. A standard result in the theory of linear systems states that in order to find the input that maximizes the output difference ||*y*||_2 _given unit input ||*u*||_2 _(so-called induced *L*_2_-norm gain), we need to find where the Bode magnitude plot, i.e., the plot of |*G*(*jω*)| versus frequency *ω*, peaks. Here, *G*(*jω*) = *C*(*jωI *- *A*)^-1^*B*, where the matrices *A*, *B *and *C *are defined in (3) and *j *denotes the imaginary unit: *j*^2 ^= -1. See [29] for more details. When the frequency at which |*G*(*jω*)| peaks is *ω*_0_, the corresponding input signal takes the form(10)

for an appropriately small *ε*, with *A *being a normalizing constant to ensure that the energy of *u*(*t*) is unit. A straightforward generalization of this concept to multiple-input multiple-output systems exists [30], which we will also use in this work.

#### Obtaining an upper bound on the L_2_-gain of the system and comparing performance

To assess the near-optimal input designed using linearization, we can compare its performance in driving the difference in the output of the two rival nonlinear models to its upper bound. This is the *L*_2_-gain of the system, for which we can again obtain an upper bound by constructing an appropriate storage function *S*. To do so, given the appropriately normalized system (2) and *ε *> 0, we assume that the trajectories with input *u *with ||*u*||_2 _= *ε *and initial condition 0 remain in a region *D *around the steady state (the origin) for all time. If there exists a *γ *> 0, and a continuously differentiable function *S*:  satisfying(11)

then

In other terms, *γ *is the desired upper bound on the maximum difference in the output of the nonlinear rival models. To see this, integrating condition (12) from 0 to *T *leads to

Therefore, for *T *→ ∞, if *x *∈ *D *for the whole time, we obtain

Here again, obtaining such a function *S *that provides the upper bound is difficult. The task of finding this bound can be relaxed to solving an SOS programme and its subsequent solution using semidefinite programming (see Methods, C).

### Design of structural changes for model discrimination

A class of experiments is based on the introduction of internal changes, such as genetic and biochemical manipulations, to the system. To mirror such experiments, we develop a methodology to pinpoint numerical changes of parameters in a system that maximize the difference between the outputs of two rival models of its internal structure. Since two such models are different, they do not necessarily have the same number of parameters. Therefore the design concentrates on the parameters that the models have in common, which we denote by *p*_*i*_, *i *= 1, ..., *m*. We assume that their values can be chosen within closed intervals [*a*_*i*_, *b*_*i*_] (where *b*_*i *_≥ *a*_*i *_≥ 0 for all *i*), that is, *p *∈ Π, where . We rewrite (2) to underline the dependence of the model on those parameters as:

and again assume that *n*_1 _= *n*_2 _= *n*, *u *= 0, *f*_1_(0, *p*) = *f*_2_(0, *p*) = 0 (for every *p *∈ Π), *h*_1_(0) = *h*_2_(0) and let

The steady state of either model may change with changing parameter *p*. Therefore the assumption *f*_1_(0, *p*) = *f*_2_(0, *p*) = 0 should be interpreted as a change of coordinates that shifts the steady state of each model to the origin individually for each *p*. We are not interested in how far the two equilibria shift *per se*, which is an algebraic problem, instead we are interested in the difference in their dynamic responses. This would reflect a situation in which a change in parameters would not be reflected in a significant change in the steady-state but which could result in a substantial difference in the dynamics of the system.

As with the previous two methods, our methodology will rely on the construction of an appropriate function *S *that sets an upper bound on the difference between the outputs of the two models, followed by a computationally efficient formulation for the construction of this function using SOS.

For the above system, suppose that there exists a function *S*: ℝ^2*n *^× Π → ℝ which is sufficiently smooth and satisfies(13)

Then(14)

if the system is released from an initial state (*x*(0), *p*) ∈ *D *× Π where *x*(0) is in a level set of *S *entirely contained in *D*, ||*x*_1_(0)|| = ||*x*_2_(0)|| = *β *≤ *α*. The last inequality in (14) holds since *S*(*x*, *p*) ≥ 0. The computational relaxation and implementation of the search for the function *S *is presented in the Methods section (section D). Once this function has been constructed, one can extract the optimal point  and parameter point  that maximizes the difference between the measured outputs of the two models.

### A case study: signal sensing in Dictyostelium discoideum

Perfect adaptation is a critical feature of many cellular signalling networks - it allows a cell to respond to a stimulus, but to re-sensitize itself so that further increases in stimulus can be detected. Adaptation is commonly used in sensory and other signalling networks to expand the input range that a circuit is able to sense, to more accurately detect changes in the input, and to maintain homeostasis in the presence of perturbations. One of the earliest examples of cellular networks exhibiting perfect adaptation is chemotaxis, which we use as a test case to illustrate our algorithms. Specifically, we use the chemotactic response in the social amœba *Dictyostelium discoideum*. Under starvation, *Dictyostelium *secretes cyclic AMP (cAMP) thus attracting other *Dictyostelium *amœbæ to aggregate and form a multicellular slug and then a fruiting body, which produces spores. Experiments indicate that a step input of chemoattractant triggers a transient response, after which the chemosensory mechanism returns to its pre-stimulus values (to its steady state), indicating perfect adaptation [31].

**Figure 1:**
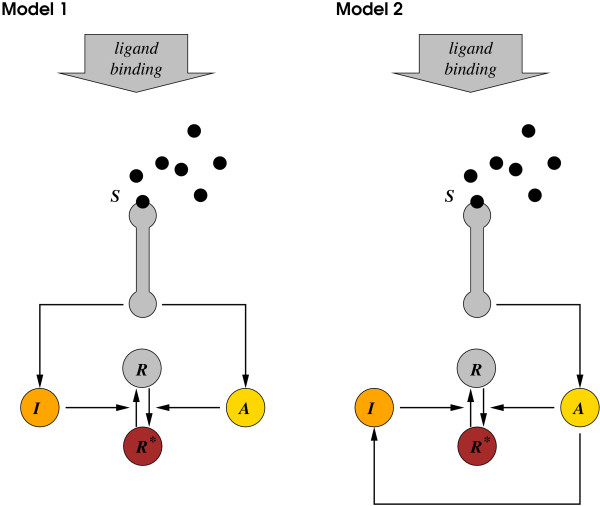
**Two models of the signal sensing system of the Dictyostelium amœba**.

At least two different simple models can describe the adaptation mechanism observed when an amœba encounters the chemoattractant cAMP [32] (Figure 1). In both models, a chemotaxis response regulator *R *becomes active (R*) through the action of an activator enzyme *A *when a cAMP ligand *S *appears. However, the deactivating mechanism determined through the interaction of an inhibiting molecule *I *in the two models can be different.

Since the sum of the concentrations of the active and inactive response regulators in the two models is constant, we can write *R*_*T *_= R*(*t*) + *R*(*t*). Consequently,  can be derived assuming mass action kinetics as:

with activation and deactivation rate constants *k*_*r *_and *k*_-*r*_.

In Model 1 both molecules, *A *and *I*, are regulated by the external signal, which is proportional to cAMP concentration *S*. With rate constants *k*_*a*_, *k*_-*a*_, *k*_-*i *_and , the dynamics of *A *and *I *are given by:

In Model 2 the inhibitory molecule *I *is activated through the indirect action of activator *A *instead of direct activation by sensing of ligand binding, giving:

where  is a rate constant. The equations for *A *are obviously identical in both models. The parameter values used are given in Table 1. Simple manipulations show that the steady-state value for R* in Model 1 is given by

while the steady-state value for R* in Model 2 is given by

Both are independent of the stimulus, explaining perfect adaptation. The two models share the same unique steady state if , a condition we impose. Note also that a value of *α *= 0.1 needs to be used in (7), as the equilibrium value for R* is at 0.1.

**Table 1:** Parameter values of the two models in the Initial condition design for model discrimination and Input design for model discrimination cases.

**Parameter**	***k*_*r*_**	***k*_-*r*_**	***k*_*a*_**	***k*_-*a*_**			***k*_-*i*_**	***S*_0_**	***R*_*T*_**
Value	1	1	3	2	1	2/3	0.1	0.2	23/30

#### Initial condition design for model discrimination

For the initial condition discriminating design, we set the input to a basal level of *S *= *S*_0_, and assume that all three concentrations, *A*, *I*, and R*, can be measured. The most discriminating initial state (*A*, *I*, R*) can be found based on the linearization of the system around its steady state using the main linear case result. The common unit length initial state which provides the direction of the perturbation from equilibrium to maximize ||*y*_1 _-*y*_2_||_2 _is then given by *x*_1_(0) = *x*_2_(0) = (1, 0, 0), where *x*_*i *_(*i *= 1, 2) are the state vectors of Model 1 and 2, respectively.

**Figure 2:**
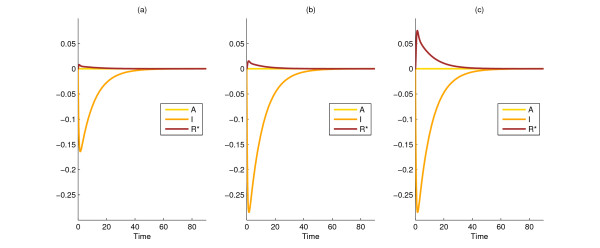
**Difference between state variables of the two rival models (states of Model 1 minus states of Model 2) in the Initial condition design for model discrimination and Design of structural changes for model discrimination cases**. Simulation results for the difference between Models 1 and 2 when started from an arbitrarily perturbed initial condition (0.5774, 0.5774, 0.5774) (a), from the 'best' unit-norm perturbation of the initial condition (1, 0, 0) (b), and with the optimal parameter changes from the corresponding optimally perturbed initial state (1, 0, 0) (c). The corresponding ||*y*_1 _- *y*_2_||_2 _values are 0.420, 0.729, and 0.747, respectively.

By applying the analogous results from the nonlinear case, the unit norm direction that maximizes the above function is also found to be *x*_1_(0) = *x*_2_(0) = (1, 0, 0), which illustrates that at least in this example, linearization can be capable of providing the correct information at a lower computational cost. Figure 2 compares the evolution of the states in Models 1 and 2 from a common arbitrary initial state (a) and the common initial state generated by the nonlinear method (b) by taking the differences between the states in the two rival models.

#### Input design for model discrimination

**Figure 3:**
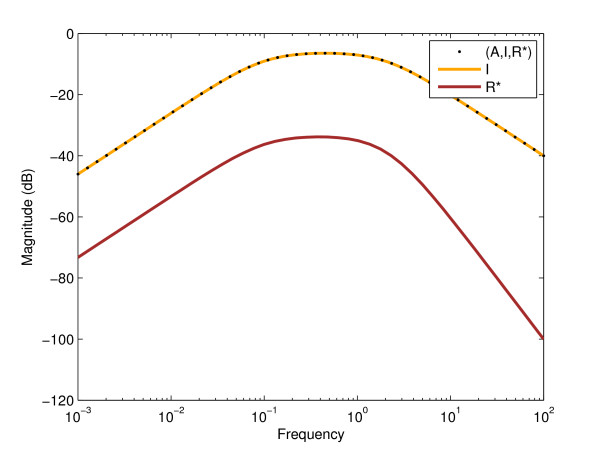
Bode magnitude plots of the linearized difference system of the two models with output (*A*, *I*, R*), *I*, and R*. The difference between the two models' R* values is marginal compared to the *I *values. The *L*_2_-gains for outputs (*A*, *I*, R*), *I*, and R* are 0.4766, 0.4762, and 0.02038, respectively. At this resolution one cannot see a difference between the cases when the output is the full state (*A*, *I*, R*) or *I *only. The shape of the Bode magnitude plot for single outputs *I *and R* is similar, with only slightly different critical frequencies (0.4472 and 0.3853, respectively). The critical frequency corresponding to completely observed state is 0.4470.

To discriminate between the two models based on an optimally chosen input profile, we first obtained an upper bound on the *L*_2_-gain of the difference system from input *S *to output (*A*, *I*, R*) using the algorithm in section C of Methods. This bound was about 0.477, a value also close to that determined through the linearization of the system (see the Bode plot shown in Figure 3).

**Figure 4:**
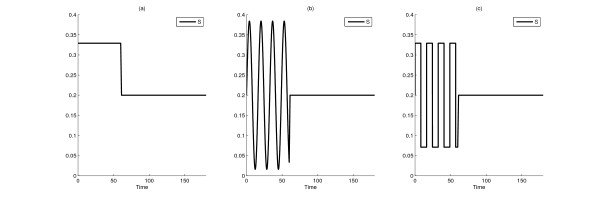
**Three distinctive input profiles *S *= *S*_0 _+ *u***. Basal input *S*_0 _perturbed until time 60 by a step function (a), a sine function (b), or a square wave function (c). ||*u*||_2 _= 1 in all cases. In (b) and (c) the frequency is 0.3853, the critical frequency corresponding to the single output R*.

In order to evaluate the performance of our algorithm, we simulated the original, nonlinear system subjected to different inputs between 0 and 60 time units including a constant, sine, cosine, sine with an exponentially increasing then decreasing multiplier, sinc, the function given by (10), or a square wave function (see Figure 4). As described earlier, for periodic inputs, the period of the input was determined by finding the frequency corresponding to the maximum amplification in the Bode magnitude plot of the linearized system (Figure 3).

**Figure 5:**
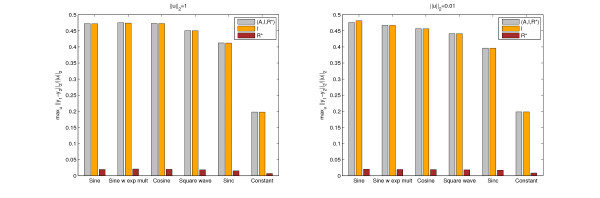
**Bar chart of data in Table 2: achievable output differences for different input profiles**.

Table 2 summarizes the results for different input perturbation functions, input perturbation energies (||*u*||_2_), and output variables using simulations of the nonlinear system. As a cosine input and the input function given by (10) (*u*(*t*) = ) gave indistinguishable results (because themselves are indistinguishable), we merged their rows into one. The top and bottom parts of the table show values for different input perturbation energies, the various columns for different output variables. The applied frequency, where relevant, was always the critical frequency corresponding to the particular output function. Figure 5 is a graphical representation of the same data.

**Table 2:** Achievable output differences for different input profiles.

||*u*||_2 _= 1	(*A*, *I*, R*)	*I*	R*
Sine	0.472	0.472	0.0195
Sine w. exp. mult.	0.475	0.474	0.0209
Cosine or (10)	0.473	0.472	0.0200
Square wave	0.451	0.450	0.0182
Sinc	0.412	0.412	0.0153
Constant	0.198	0.197	0.0070

**||*u*||_2 _= 0.01**	(*A*, *I*, R*)	***I***	R*

Sine	0.476	0.481	0.0203
Sine w. exp. mult.	0.467	0.467	0.0195
Cosine or (10)	0.457	0.456	0.0191
Square wave	0.441	0.441	0.0184
Sinc	0.396	0.396	0.0173
Constant	0.198	0.198	0.0085

Numerical estimates of max_*u *_||*y*_1 _- *y*_2_||_2_/||*u*||_2 _with different inputs for the *Dictyostelium *models by simulating the nonlinear system. Here maximization is over different frequencies for inputs where frequency makes sense. Output is either all states or *I *or R*.

**Figure 6:**
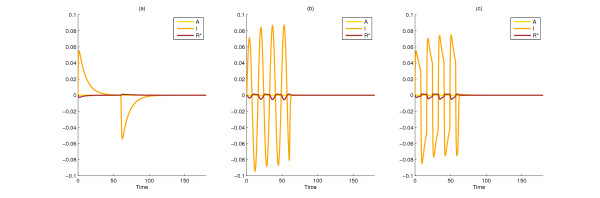
**Difference between state variables of the two rival models (states of Model 1 minus states of Model 2) in the Input design for model discrimination case**. Simulation results for Models 1 and 2 with a constant (a), sine (b), or square wave (c) perturbation of the basal input *S*_0 _until time 60 (the inputs in Figure 4). The output variable (on which the choice of the optimal frequency depends) is R* and ||*u*||_2 _= 1. The corresponding ||*y*_1 _- *y*_2_||_2 _values are 0.0070, 0.0195, and 0.0182, respectively. Note that the input signal *S*_0 _+ *u *is not included in the figure.

Figure 6 compares the differences in state variables between the two models for three typical input perturbations. Interestingly, our results indicate that for all inputs used, discrimination between the two models should be accomplished by measuring output *I *rather than output R* (also seen in Table 2). In the first plot, the basal input *S*_0 _is perturbed by a step function between 0 and 60 time units (Figure 4a). In the second plot, the system is injected with a sine function (Figure 4b). In the third plot, the input is a square wave function (Figure 4c), a caricature of the sine function with preserved period that can be realized in practice more easily. The sine input yields a visibly larger difference than the step function between the R* values of the two models. The square wave function produces a similarly good result. One has to note that one measurement may not be enough for the discrimination, but a series of measurements may be needed.

Perhaps the most notable outcome of the input design is that sinusoidal input perturbations generate the best *L*_2_-gains and are therefore superior to a step function for discriminating between rival chemotaxis models. Square wave stimulation is achievable in the reality of a laboratory. This is important since step inputs are usually used in experiments, often at the exclusion of other input signals. Our studies demonstrate how more dynamic inputs, in this case an oscillating input (on a finite time interval), might be necessary to delineate subtle features of underlying network topologies.

#### Design of structural changes for model discrimination

For the design of most informative structural and parameter perturbations, the equilibrium is dependent on the particular choice of parameters. Therefore, we illustrate in detail how to change coordinates in order to translate the equilibrium to zero. Let the state variables for Model 1 be *x*_11 _= *A*, *x*_12 _= *I*, and *x*_13 _= R* and those for Model 2 be *x*_21 _= *A*, *x*_22 _= *I*, and *x*_23 _= R*. Assume that in both models the parameters that can be modified before the experiment are *R*_*T*_, the total chemotaxis response regulator concentration and *p *= *k*_*r*_, the response regulator activation rate constant. Parameter values and the intervals of values that can be achieved are given in Table 3. The dynamics of the two models are given by

The steady states for these equations are given by

Let , *i *= 1, 2, *j *= 1, 2, 3. We can now perform a change of coordinates to make the origin the equilibrium point of:

**Table 3:** Parameter ranges of the two models in the Design of structural changes for model discrimination case.

**Parameter**	***k*_*r*_**	***k*_-*r*_**	***k*_*a*_**	***k*_-*a*_**			***k*_-*i*_**	***S*_0_**	***R*_*T*_**
Range or value	[0.5, 1.5]	1	3	2	1	2/3	0.1	0.2	[0.5, 3.0]

In order to discriminate between Models 1 and 2, we first solve the optimization programmes given in section D of Methods, as explained in the section on *Design of structural changes for model discrimination*. We allow parameters *R*_*T *_and *k*_*r *_to vary. We obtain that (0) = (0) = (1, 0, 0) for the initial conditions, and *R*_*T *_= 3 and *k*_*r *_= *p *= 1.5 for the values of the parameters that have maximal discriminating power between the two models. (See Figure 2c.). This means that we need to over-express the total number of chemotaxis response regulators and increase their rate of activation in order to see a large difference between the two models.

The optimization problems in all three cases were solved on a desktop computer. The most challenging was the first SOS programme for the last case with eight variables (three state variables for each model and two parameters). Numerical methods will need to be improved in order to deal with SOS programmes resulting from the analysis of more complex systems biological models.

## Conclusions

In this paper we have developed methods for designing experiments to effectively discriminate between different models of a biological system. These methods are tailored to generate maximally informative data that can be used to invalidate models of gene regulatory pathways by ruling out certain connectivities in their underlying biochemical reaction networks [15]. We approached the problem in a unified framework, developing methodologies for initial conditions design (see also [23]), for the design of dynamic stimulus profiles, and for parameter modifications. These types of manipulations cover a large spectrum of what is experimentally feasible, and this has largely informed our formulation of the problem and the approach to its investigation.

If the field of systems biology is to accelerate the pace of biological discovery, rigorous mathematical methods should be developed to link computational models of biological networks to experimental data in tight rounds of analysis and synthesis. Any informative model should be analyzed in light of existing data, but it should also be able to synthesize new experiments that further delineate the features of the underlying biological system. Despite many notable examples demonstrating the success of this iterative procedure, progress has been slow due to the *ad hoc *nature of its implementation: the iterations between the development of models and the production of data is still mostly guided by the intuition of the modellers, and no rigorous algorithms exist to render this process more systematic and less biased. We believe that the work presented in this paper constitutes an important step in this direction. By design, our formulation of the problem is of sufficient generality to accommodate many experiment design procedures, and is cast in a natural optimization framework. Acknowledging that optimality of experiment designs must always be balanced with biological and other practical constraints, our formalisms allow for the incorporation of limitation and constraints as dictated by the specific biological context. For example, if demanding a sinusoidal input may be unrealistic in a laboratory setting, and an optimal input in a smaller input function space is practically required, such constraints can be added to the nonlinear optimization criteria.

We illustrated the applicability of our algorithms using two possible and widely accepted simplified models of the adaptation mechanism in *Dictyostelium discoideum *chemotaxis. Evidently, these models do not capture the full complexity of the biological circuit responsible for chemotactic behaviour. The models, however, illustrate the core circuit topologies that are sufficient to implement perfect adaptation in the system. Recent work investigating perfect adaptation demonstrated that despite the diversity of biochemical enzymatic networks, only a finite set of core circuits with defined topological features can execute a desired function [33]. These findings highlight the possibility of distinguishing between mechanisms that implement a given biological function using simple models, empowered by model-discrimination methods such as those presented in this work. We also applied the optimal experiment design methods described in this paper to invalidate models of the chemotaxis pathway in *Rhodobacter sphaeroides *[34]. There, the combination of a square wave profile stimulation and protein over-expression was necessary in the most challenging model discrimination problem. This demonstrates the practical demand for sophisticated experiment design techniques.

The recipe for model discrimination that we propose involves collecting mostly time series data. Every new time point at which measurements are made increases the cost of experiments, and thus one must carefully balance the number of time points collected against the cost, and consider where along a time series to concentrate observations. Our methods naturally present a window into this question by providing the timescales at which data collection needs to be done to be maximally informative. Furthermore, if the optimal experiment is such that a differentiating dynamical phenotype only emerges several hours after a perturbation, our methods can be easily modified to balance optimality with practically measurable dynamics.

Finally, many commonly used perturbations (genetic or environmental) lead to either extreme stress responses that put a cell in a modified physiological state, cell death, or quiescent states that do not have much measurable information about the underlying regulatory network. Experiments that generate less catastrophic failures of cellular networks under study, while being maximally informative, hold great promise for the study of biological networks. Finding this region in perturbation space, however, is a nontrivial task. Model-based design of experiments will undeniably be instrumental for that, ultimately leading to many important biological discoveries.

## Methods

### A - Sum of squares (SOS) decompositions

Here we present the *sum of squares *formalism which is used to relax and solve the optimization problems posed by the various approaches for model discrimination considered in this paper.

A polynomial *p*(*y*) in *y *= (*y*_1_, ..., *y*_*n*_) with real coefficients is nonnegative if *p*(*y*) ≥ 0 for all *y*. It is a *sum of squares *(SOS) if there exist other polynomials *p*_*i*_(*y*), *i *= 1, ..., *M *such that . Obviously, such a polynomial is nonnegative, but the converse is not always true [24]. In fact, testing if *p*(*y*) ≥ 0 is NP-hard [35], but testing if *p*(*y*) is a sum of squares is equivalent to a so-called semidefinite programme (SDP) [24], a convex optimization problem for which there are algorithms that can solve it with a worst-case polynomial-time complexity. SOSTOOLS [36] can be used to formulate this SDP which can be solved using SDP solvers such as SeDuMi [37] or SDPT3 [38].

### B - SOS programme for initial condition design

The strategy outlined in the section on *Initial condition design for model discrimination *relies on the construction of a function *S *satisfying the nonnegativity conditions given by (5-6). For reasons pointed out in section A, constructing a nonnegative *S *is difficult. We therefore relax nonnegativity to the existence of an SOS decomposition and solve the problem through semidefinite programming. An SOS programme that can be used to generate *S *is

The last constraint ensures that *f*(*x*) -*h*^*T*^*h *≥ 0 when *x *∈ *D*, since the multipliers *σ*_1_(*x*_1_) and *σ*_2_(*x*_2_) are SOS. The solution *S *is not unique, but a heuristic to find the 'best' *S *is to optimize over the decision variables in the SOS description for *S *(by minimizing the trace of the Jacobian of *S *at the origin), so that the resulting *S *has sub-level sets that have maximal area.

In the second step we solve the SOS relaxation of the optimization problem (9) to get the initial state *x*_1_(0) = *x*_2_(0) = :

The point  can be obtained from the dual solution of this semidefinite programme, using SOSTOOLS.

### C - SOS programme for optimal input design

In the *Input design for model discrimination*, we should first note that it may occasionally be the case that the set of inputs considered will lead to a system trajectory outside the region where *S *is constructed. This case can be ruled out by solving a related reachability problem [39]. Here, we assume that the containment of the trajectory in *D *has been ensured, and describe how to obtain an estimate of the *L*_2_-gain of the system.

To construct a function *S *which satisfies the conditions shown in the section on *Input design for model discrimination*, we use the SOS framework as follows. Condition (12) can be satisfied by searching for SOS multipliers *σ*_1_(*x*) and *σ*_2_(*x*) such that

where *α *> 0 is used to define the region *D *by

This condition guarantees that *f*(*x*) - *y*^*T*^*y *+ *γu*^*T*^*u *≥ 0 for *x *∈ *D*. The rest of the conditions can also be easily enforced in an SOS programming framework.

Consequently, the overall SOS programme for constructing *S *takes the form:

### D - SOS programme for optimal structural design

The search for a function *S*(*x*, *p*) ≥ 0 such that (13) holds in *D *× Π can be formulated as:

As in the initial state and input design cases, we would like to maximize the difference given by *y*. Although we cannot achieve this goal directly, we can maximize the approximation of  given by *S*(*x*(0), *p*) (see (14)), where we require that *x*_1_(0) = *x*_2_(0) =  and ||||_2 _= *β*. We introduce the modified *S*, . Similarly, . The problem is then the following.

Exactly as in the initial state design case, the point  can be obtained from the dual solution, using SOSTOOLS.

## List of abbreviations

cAMP: cyclic adenosine monophosphate; SDP: semidefinite programme; SOS: sum of squares; s.t.: subject to.

## Authors' contributions

BM developed the second approach and wrote the first draft of the manuscript. EA developed the third approach. AP developed the first approach together with HE, participated in the development of the other two approaches and finalized the manuscript. All authors read and approved the final manuscript.
